# Estimation of Recurrence Interval of Large Earthquakes on the Central Longmen Shan Fault Zone Based on Seismic Moment Accumulation/Release Model

**DOI:** 10.1155/2013/458341

**Published:** 2013-06-26

**Authors:** Junjie Ren, Shimin Zhang

**Affiliations:** Key Laboratory of Crustal Dynamics, Institute of Crustal Dynamics, China Earthquake Administration, Beijing 100085, China

## Abstract

Recurrence interval of large earthquake on an active fault zone is an important parameter in assessing seismic hazard. The 2008 Wenchuan earthquake (Mw 7.9) occurred on the central Longmen Shan fault zone and ruptured the Yingxiu-Beichuan fault (YBF) and the Guanxian-Jiangyou fault (GJF). However, there is a considerable discrepancy among recurrence intervals of large earthquake in preseismic and postseismic estimates based on slip rate and paleoseismologic results. Post-seismic trenches showed that the central Longmen Shan fault zone probably undertakes an event similar to the 2008 quake, suggesting a characteristic earthquake model. In this paper, we use the published seismogenic model of the 2008 earthquake based on Global Positioning System (GPS) and Interferometric Synthetic Aperture Radar (InSAR) data and construct a characteristic seismic moment accumulation/release model to estimate recurrence interval of large earthquakes on the central Longmen Shan fault zone. Our results show that the seismogenic zone accommodates a moment rate of (2.7 ± 0.3) × 10^17^ N m/yr, and a recurrence interval of 3900 ± 400 yrs is necessary for accumulation of strain energy equivalent to the 2008 earthquake. This study provides a preferred interval estimation of large earthquakes for seismic hazard analysis in the Longmen Shan region.

## 1. Introduction

Recurrence interval of large earthquakes, closely associated with dynamic process of the seismogenic fault, is a key parameter for seismic hazard assessment on a seismic fault, especially on large-scale fault zones [1, 2]. The 2008 Wenchuan earthquake (Mw 7.9) broke the central Longmen Shan fault zone and caused the loss of large property and many thousands of lives [3, 4]. It ruptured the YBF and GJF [4]. The most concern for local residents is how often large earthquakes occur on the Longmen Shan fault zone.

**Figure 1:**
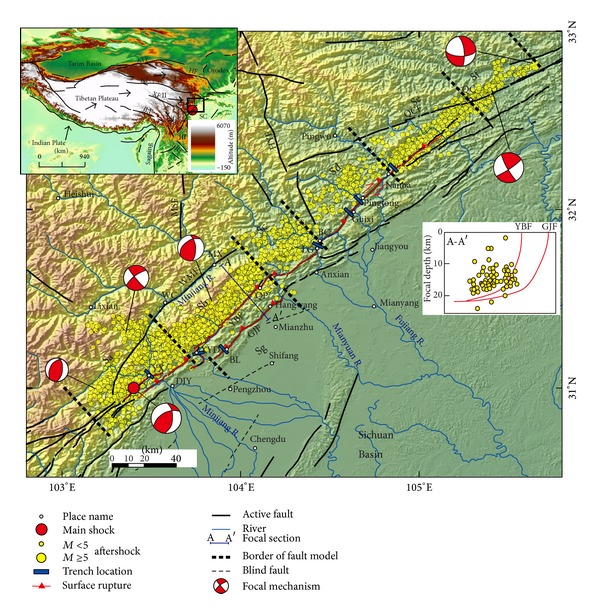
Tectonic settings and aftershock sequence of the 2008 Wenchuan earthquake. Surface rupture is modified from [4]; focal mechanisms are from USGS; aftershock sequence is from relocated results [28]. Borders of fault model are according to GPS and InSAR inversion [6]. Trench sites are from published results [12–14]. White circles are place names: BC, Beichuan county; BL, Bailu town; DJY, Dujiangyan city, LG, Leigu town; MX, Maoxian county; QC, Qingchuan county; QP, Qingping town; WC, Wenchuan county; XYD, Xiaoyudong town; YX, Yingxiu town. Black solid lines are known faults: YBF, Yingxiu-Beichuan fault; GJF, Guanxian-Jiangyou fault; WMF, Wenchuan-Maoxian fault; QCF, Qingchuan fault; MSF, Minshan fault; HYF, Huya fault. Insert map shows the topography of the Tibetan plateau. Inset map shows major tectonics in the Longmen Shan vicinity: ATF, Altyn Tagh fault; HF, Haiyuan fault; JLF, Jiali fault; KF, Kunlun fault; RF, Red River fault; XF, Xiaoshuihe fault; I, Qaidam-Qilian block; II, Bayan Har block; III, Sichuan-Yunan block. Black arrows indicate block motion direction.

Several methods are used to estimate recurrence interval of large earthquakes on the central Longmen Shan fault zone. Based on the ratio of coseismic displacement and long-term GPS/geologic slip rate, the average recurrence intervals for large earthquakes were roughly estimated to 2000–10000 yrs [5], 3000–6000 yrs [3], and ~4000 yrs [6]. However, this method includes many uncertainties due to the homogeneity of coseismic slip along the fault trace [4, 7, 8]. Paleoseismologic excavation is direct and effective to obtain the interval of large earthquakes [2]. Due to limitations and uncertainties of dating techniques, however, the results of different authors reveal a remarkable discrepancy of recurrence interval, even in the same trench. Preseismic trenches at Leigu and Baishuihe towns along the YBF showed that the penultimate event occurred at 13.81–11.77 ka, based on radiocarbon dating [9, 10]. In the same Qingshiping trench, Li et al. and Densmore et al. suggested the interval of 3830 and 930 yrs, respectively, based on radiocarbon dating along the GJF [10–12]. Many trenches have been excavated across the coseismic surface rupture following the 2008 earthquake (Figure 1). Some trenches were emplaced at Yingxiu, Xiaoyudong, Leigu, and Pingtong towns along the BYF and others, at Bailu town along the GJF (Figure 1). Lin et al. proposed a recurrence interval of ~1000–1200 yrs in the Yingxiu and Leigu trenches based on archaeological evidence and radiocarbon dating [13]. The trenches at Yingxiu, Xiaoyudong, Leigu, and Bailu towns using radiocarbon dating showed a recurrence interval of 2300–3300 yrs [14, 15]. The trenches of Li et al. at Pingtong, Guixi, and Nanba towns from optically stimulated luminescence (OSL) and radiocarbon dating revealed a ~11000 yr interval [12]. Liu et al. advised a recurrence interval of 1100–2100 yrs in the Leigu trench utilizing OSL and radiocarbon dating [16]. However, Wen et al. recommended that the recurrence interval of large earthquakes in and adjacent to the 2008 epicenter area is longer than 2000 yrs according to historical earthquake activity [17].

Significant discrepancies between these results confuse the public that which value is preferred for large earthquake on the Longmen Shan fault zone. In this paper, we construct a characteristic seismic moment accumulation/release model, based on fault geometry of the seismogenic zone and estimate the average recurrence interval of earthquakes similar to the 2008 shock along the central Longmen Shan fault zone.

## 2. Characteristic Seismic Moment Accumulation/Release Model

Seismic activity is a process of energy accumulation and release [18]. Seismic moment is a measure of the size of an earthquake in terms of the energy released and is associated with the seismogenic zone on a fault [19]. The characteristic seismic moment accumulation/release model agrees with the energy balance principle and assumes that the moment released by an earthquake is equal to the accumulation along a seismic fault during an recurrence interval.

If the mean moment of repeated earthquakes, *M*_0_ (in N m), and the long-term moment accumulation rate on the seismic fault, ${\overset{-}{M}}_{0}$ (in N m/yr), are known, their ratio would define the average recurrence interval of earthquakes, *T*  (in yr) [20]:

(1) $$\begin{array}{c} T=\frac{M_{0}}{{\overset{-}{M}}_{0}}. \end{array}$$

For modern earthquakes, seismic moment (*M*_0_) is usually estimated from seismograms. Seismic moment can also be converted from the moment magnitude (*M*_*w*_) using the formula of Hanks and Kanamori [21].

The long-term moment accumulation rate on each fault segment is represented as a rectangular fault patch with uniform secular slip rate. The moment rate of each segment (${\overset{-}{M}}_{0s}$) is obtained from seismogenic area (*A*) in km^2^ and long-term slip rate (*ν*) in mm/yr:

(2) $$\begin{array}{c} {\overset{-}{M}}_{0s}=\mu A\nu , \end{array}$$

where  *μ* is the shear modulus.

**Figure 2:**
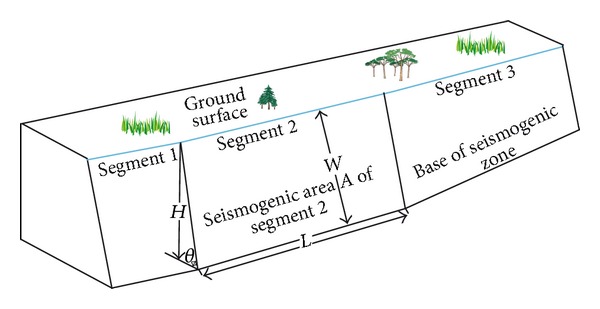
Conceptual illustration of a segmented oblique fault. Also shown are measures of length *L*, down-dip width *W*, epicenter depth *H*, and dip angle *θ*.

The seismogenic area (*A*) is the area of fault plane ruptured in an earthquake (Figure 2):

(3) $$\begin{array}{c} A=LWR, \end{array}$$

where  *L* is segment length, the distance between two segmentation points. *W* is down-dip segment width, corresponding to the thickness of the brittle upper crust in which strain energy available to be released as earthquakes is stored. If the fault plane is oblique, *W*  is the ratio of the epicenter depth (*H*) and the sine of dip angle (*θ*): *W* = *H*/sin(*θ*). If the fault plane is vertical, *W* is equal to the focal depth (*H*). *R* is a slip scaling factor (ranging from 0 to 1) that accounts for the role of fault creep in reducing the fault slip available for earthquake rupture and varies from *R* = 0 (all slip occurs aseismically) to *R* = 1 (all slip occurs in earthquakes).

Then, the moment rate along each fault segment (${\overset{-}{M}}_{0s}$) can be defined by the formula:

(4) $$\begin{array}{c} {\overset{-}{M}}_{0s}=\frac{\mu LHR\nu }{\sin\left(\theta \right)}. \end{array}$$

So the moment rate of the total seismogenic zone is the sum of all the segments:

(5) $$\begin{array}{c} {\overset{-}{M}}_{0}=\sum {\overset{-}{M}}_{0s}. \end{array}$$

Assume that similar-size large earthquakes or characteristic earthquakes always occurred along a seismic fault and the moment accumulation rate is constant, then Formula (1) can be used to approximately estimate recurrence interval of large earthquake on this fault.

## 3. Tectonic Settings of the 2008 Wenchuan Earthquake

The 2008 Wenchuan earthquake occurred on the central part of the northeast-trending Longmen Shan thrust belt, which bounds the eastern margin of the Tibetan plateau and is characterized by the steepest relief along any margin of the Tibetan plateau [5]. The Longmen Shan thrust belt is ~500 km long and consists of three main subparallel thrust faults: the Wenchuan-Maoxian fault, YBF, and GJF (Figure 1), which merged at the basal detachment ~15–20 km deep and formed an imbricated thrust fault system [22]. Crustal shortening on this system is the possible main cause for uplift of eastern Tibet [23].

 The 2008 earthquake ruptured the central Longmen Shan fault zone and generated the ~240 km and ~70 km surface rupture along the YBF and GJF, respectively [4]. The coseismic surface rupture is mainly expressed by thrust slip in the south and equivalent strike and thrust slip components in the north [4, 24]. Seismic-wave inversion showed that a ~300 km-long rupture in the seismogenic zone was generated during the 2008 earthquake [25].

## 4. Seismic Moment Released in a Recurrence Cycle

Seismic moment is released in a recurrence cycle including main shocks, aftershocks, and interseismic earthquakes. The seismic moment released by the 2008 main shock and some aftershocks has been calculated from the amplitude spectra of seismic waves [26]. Other earthquakes only has surface-wave magnitudes. Hence, the conversion relationship between scalar moment and surface-wave magnitude in the Longmen Shan area is required for our analysis.

**Figure 3:**
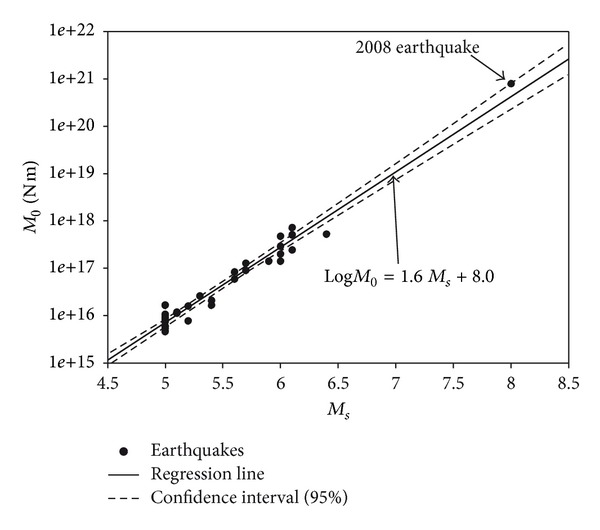
Regression line of seismic moment (*M*_0_) and surface magnitude (*M*_*s*_). Data include the main shock and 33 aftershocks with *M*_*s*_ ≥ 5.0 during the 2008 earthquake [43].

Zheng et al. estimated scalar seismic moment of the main shock and 33 aftershocks bigger than *M*_*s*_ 5.0 based on waveform data from National Digital Seismograph Network and regional seismograph network of China [26]. All the 34 samples are used to establish the regression relationship of surface-wave magnitude (*M*_*s*_) versus scalar seismic moment (in N m) by least-squares fitting (Figure 3):

(6) $$\begin{array}{c} \text{Log}M_{0}=1.6M_{s}+8.0. \end{array}$$

The correlation coefficient is 0.98.

From 12 May 2008 to 1 December 2012, there are 749 *M*_*s*_ ≥ 4.0 aftershocks occurred. Before the 2008 earthquake, seismic activity in historical and instrumental records merely includes four moderately strong earthquakes, the 1597 *M*_*s*_ 5.0, 1913 *M*_*s*_ 5.0, 1958 *M*_*s*_ 6.2, and 1999 *M*_*s*_ 5.4 earthquakes [17]. Using formula (6), the scalar moment released by *M*_*s*_ ≥ 4.0 aftershocks and interseismic earthquakes is 2.05 × 10^19^ N m. The 2008 main shock is equivalent to Mw 7.9 according to USGS and China Earthquake Administration, and the released scalar moment is 1.04 × 10^21^ N m using the conversion formula [21]. So, the scalar seismic moment release in a whole recurrence cycle is 1.06 × 10^21^ N m, showing that aftershocks and moderate earthquakes play a minor role (~2%) in the release of seismic moment along the central Longmen Shan fault zone.

## 5. Moment Accumulation Rate in the Seismogenic Zone

### 5.1. Fault Geometry

Inversion of seismic waves and field investigation suggested that the seismogenic zone of the 2008 earthquake can be divided into several segments, which represents a complicated rupture process in this earthquake [4, 25, 27]. Detailed fault geometry of the seismogenic zone associated with this earthquake was modeled based on GPS and InSAR data [6]. In this fault model, the BYF dips to the northwest at a moderate dip of ~43° at the southwest end of the rupture belt, reaching ~50° at Nanba. The fault has a dip of ~56° across the Nanba step-over and increases progressively to near vertical at the northeast terminal of the rupture. The GJF generally dips ~28°.

### 5.2. Focal Depth (*H*)

**Figure 4:**
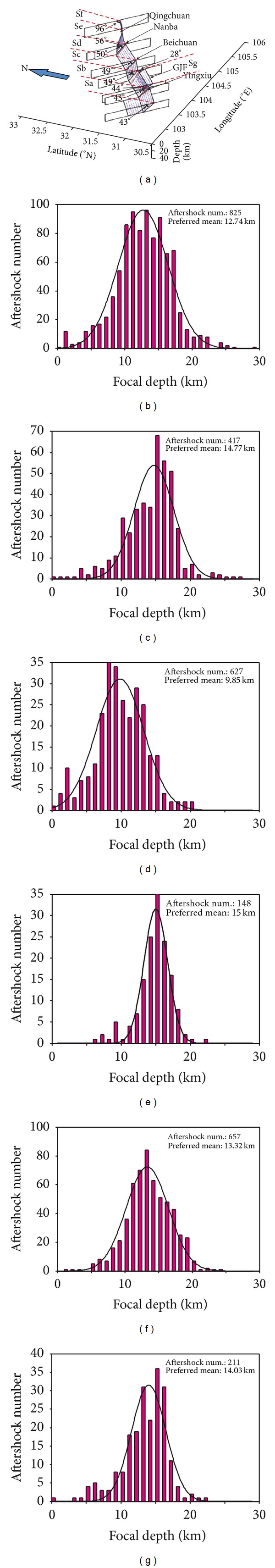
Fault model and focal depth of the seismogenic zone along the central Longmen Shan fault zone. (a) Fault geometry and segments viewed from the southwest, at 45° elevation angle (modified from [6]). Borders of fault segmentation are shown in Figure 1. (b)–(g) are statistic results of focal depth for segments Sa–Sf, respectively, according to the relocated aftershocks [28]. Statistical samples of each segment are aftershocks enclosed by segment borders, as shown in Figure 1. Black solid lines are regression curves of Gaussian distribution, which pass the Shapiro-Wilk Normality test. The value of preferred mean is estimated from the regression result.

The seismogenic zone associated with the 2008 earthquake is complex, and each segment has a different focal depth. According to the fault model [6], six segments (Sa–Sf) are divided along the YBF, and a separate patch (Sg) is along the GJF (Figure 4(a), Table 1).

The 2553 aftershocks, from May 12 to July 8, 2008, were relocated according to different velocity models for the east and west side of the Longmen Shan fault zone [28], using double-difference algorithm [29]. To estimate the preferred seismogenic depth of each segment, the frequency histogram of aftershock focal depth and the regression curve of Gaussian distribution in each segment are made. The regression results pass the Shapiro-Wilk Normality test. Preferred depth of each segment is estimated from the regression mean (Figures 4(b)–4(g),Table 1).

Seismic reflection data and well logs indicate that the YBF and GJF merge at the base of the seismogenic zone, which appears to root into a detachment in the mid-crust [22, 23, 30, 31]. The lack of aftershocks and a shallow dip angle along the Sg segment suggest a common root shared with the Sb segment of the BYF at the seismogenic depth, a result in agreement with balanced geological cross-sections across the southern Longmen Shan thrust zone [23, 30]. Therefore, the focal depth of the Sb segment is assigned to the Sg segment (Table 1).

### 5.3. Slip Rates along the Central Longmen Shan Fault

Fault slip rate can be derived from geological field investigation and geodesy. Geological rate is an average value of long-term tectonic movement and is constraint by dated offset marker units, while geodetic fault slip rate estimates are based on model based inferences from interseismic velocity gradients. From a long-term respective, both slip rates can be approximately equivalent [32].

Geological observation and GPS surveys show a relatively low slip rate along the Longmen Shan thrust zone and some differences between geological and GPS rates (Table 1).Given the uncertainties in the estimation of geological rates, GPS slip rates are preferred in our calculations. For segments Sb and Sg where there is a lack of GPS result, geological rates are used.

### 5.4. Moment Accumulation Rate

To estimate moment accumulation rate on all the segments, we integrate the thrust and dextral slip rate components along down-dip and strike. Using formula (4), moment accumulation rate of each segment is calculated in Table 1, assuming a shear modulus of *μ* = 30 GPa in the crust and a slip scaling factor *R* of 0.9 given large thrust slip on the Longmen Shan fault belt. The total moment accumulation rate of the seismogenic zone is 2.67 × 10^17^ N m/yr using formula (5).

Model uncertainty is prevalent in estimating the recurrence interval because the characteristic moment accumulation/release model depends on some assumptions and uncertain input parameters. 

In our model, slip rate on each fault segment is assumed to be constant with time. Although some fault zones have been found to change of slip rate during the late Quaternary [33–35], slip rate along the Longmen Shan fault belt is roughly comparable (Table 1). 

Other assumption is moment balancing, which demands that all the moments accumulated in the interseismic period are released by the earthquakes in a recurrence cycle. The central Longmen Shan thrust belts are composed of not only the three main faults but also some blind thrust faults in the Sichuan basin as shown in Figure 1. Postseismic investigations showed that the 2008 earthquake also formed small folds (~10–20 cm) and coseismic cracks on the ground surface in the Sichuan basin [36], which suggests a small portion of moment may be released by these blind thrust faults in the basin. In addition, although postseismic deformation, moderate and small earthquakes, and the influence of other adjacent large earthquakes exist on the Longmen Shan fault belts [37, 38], they play a minor role in strain accommodation in a recurrence interval. 

Slip rate is a crucial input parameter in our model. Continuous GPS survey across the fault zone provides an accurate slip rate [39]. However, along some parts of fault zone, there is a lack of continuous GPS stations. In the estimation of geological rate, accurate offset and formation age of geomorphic surface are required [2]. An accurate offset demands a clear geomorphologic marker. Owing to numerous external factors, erosion, human activity, and so on, these markers are prone to be destroyed and illegible. Age of geomorphic surface needs a sample that presents the formation and an appropriate dating technique. Various dating methods have their own limitations. For example, OSL demands a bleached sufficiently luminescence signal, and radiocarbon needs autochthonous carbonaceous material and to be well-preserved (i.e., not obviously contaminated with carbon not original to itself ) [40].

 The slip scaling factor *R* is another parameter that influences the moment rate and represents the role of fault creep in the release of seismic moment. Although fault creep is found on numerous faults, especially on large-scale strike-slip faults [41], there is lack of detailed study about the *R*-factor on the Longmen Shan fault zone. In this calculation, the *R* value of 0.9 is adopted because of more difficulties of creep slip on thrust faults than on strike-slip and normal faults. Whether this value is suitable for the Longmen Shan fault zone needs further work.

The uncertainties in all the parameters involved in this calculation are hard to be determined precisely. The area of seismogenic zone and slip rate along the fault segments generally have a 10% uncertainty in the rupture model [6], respectively. Thus, we infer that ~20% uncertainty should be considered in our model. So the total moment accumulation rate of the seismogenic zone is (2.7 ± 0.3) × 10^17^ N m/yr in the seismogenic zone.

## 6. Recurrence Behavior and Interval of Large Earthquakes

Postseismic trenches at Yingxiu, Xiaoyudong, Leigu, Pingtong, Guixi, Nanba, and Bailu towns along the 2008 coseismic surface rupture (Figure 1) revealed that the penultimate large earthquake had a coseismic offset similar to the 2008 earthquake [10–15], consistent with the result of offset geomorphology [27]. In other word, the central Longmen Shan fault zone probably undertakes the repeated earthquake similar to Mw 7.9, consistent with a characteristic earthquake model [42]. Small and moderate earthquakes might probably be background earthquakes in a recurrence cycle. Using formula (1), the average recurrence interval of large earthquakes similar to the 2008 earthquake along the central Longmen Shan fault belt is 3900 ± 400 yrs.

In addition, China has a long record of earthquake, especially for Xi'an city, ever the capital of several dynasties in the Chinese history and is ~400 km away from the 2008 surface rupture and felt strong motion in the 2008 earthquake. The city began to have a detailed document since at least 316 BC (the age of the reign of Qin Dynasty). The earliest written literature on earthquakes was a felt earthquake occurring at 263 AD [43]. Since that time, the catalog of large earthquakes is probably complete. Dujiangyan weir, located in Dujiangyan County, west Chengdu City and only several kilometers away from the 2008 surface rupture (Figure 1), is a famous water conservation project and was constructed in the Qin Dynasty (~200 BC). Since that time, Chengdu, ever the capital of Shu Kingdom in ancient China, has become the most important economic and political center. If a large earthquake similar to Mw ~7.9 even occurred in the Longmen Shan region, the county annals of adjacent areas, like Chengdu and Xi'an cities, would give a literature record about this quake. According to the documents of historical earthquakes, there is a lack of great earthquake in the 2008 epicenter area and adjacent regions [43]. To sum up, the recurrence interval of large earthquakes along the central Longmen Shan fault zone is greater than ~2300 yrs.

The recurrence intervals of Densmore et al. [11], Lin et al. [13], and Liu et al. [16] are shorter than the time span of earthquake record. The main reason could be as follows. The archaeological materials found in the trench might be related to postearthquake human activity and not to the penultimate earthquakes. The ages of radiocarbon dating are younger due to polluted samples by newer carbonaceous matters [2, 44]. The result of Li et al. is apparently older than the time span of earthquake record [12]. The reason could be that the OSL samples of colluvium related to the earthquake might not have been bleached sufficiently [45].

Our result is probably not a real value but a preferred estimate of average interval. The real recurrence interval needs further carefully dating of trench sampling. This value will provide a relatively reasonable recurrence interval for the central Longmen Shan fault zone.

As the boundary fault zone between the Tibetan plateau and Sichuan basin, the Longmen Shan thrust zone has accommodated the strain from eastward extrusion of the Tibetan plateau. The strain is released by seismic slip along thrust faults within the Longmen Shan thrust fault zone and exhibited by great relief. However, geodetic measurements and geomorphic investigations show that east-west shortening across the range is relatively small (<3 mm/yr) [10, 39, 46, 47], in comparison with that of other border faults around the Tibetan plateau. This limited rate might need a millennial-scale interval to accumulate the strain energy equivalent to the 2008 earthquake.

## 7. Conclusions

Paleoseismological excavations following the 2008 earthquake show that the similar-size earthquake probably always rupture the YBF and GJF, which suggests that the central Longmen Shan fault zone accords with a characteristic earthquake model equivalent to Mw 7.9 [12–15]. 

Seismic moment release indicates that the moment on the central Longmen Shan fault zone is probably released by large earthquakes, and the role of small and moderate earthquakes is minor. Based on the characteristic seismic moment accumulation/release model, the YBF and GJF accommodate a moment accumulation rate of (2.7 ± 0.3) × 10^17^ N m/yr, and the Mw 7.9 earthquake needs an interval of 3900 ± 400 yrs to accumulate energy on the central Longmen Shan fault zone. 

## Figures and Tables

**Table 1:** Parameters of fault model and accumulation moment rate in the seismogenic subsegments.

Seismogenic zone				Dextral rate (GPS, Geol) (mm/yr)	Reverse rate (GPS, Geol) (mm/yr)	Secular Rate (GPS, Geol) (mm/yr)	Moment rate (10^16^ N m/yr)
Segment	Length (km)	Dip (°)	Depth (km)				
Sa	68	43	12.74	1.7, 1.0	1.4, 0.3–0.6	2.2, 1.2	8.38
Sb	62	44	14.77	/, 1.3	/, 0.54	/, 1.41	4.75
Sc	41	49	9.85	1.7, 0.96	1.4, 1.1	2.2, 1.2	3.53
Sd	51	50	15.00	0.8, /	0.3, /	0.85, /	2.40
Se	60	56	13.32	0.8, /	0.3, /	0.85, /	2.31
Sf	47	90	14.03	0.8, /	0.3, /	0.85, /	1.58
Sg	63	28	14.77	/, 0.89	/, 0.23	/, 0.92	1.19

Dip angles and GPS rates are from [6, 39], as shown in Figure 3; geological rates are from [9–11, 46]; depth values are from Figure 4. Moment rate is calculated using formula (4).
